# A multi-laboratory profile of *Mycoplasma *contamination in *Lawsonia intracellularis *cultures

**DOI:** 10.1186/1756-0500-5-78

**Published:** 2012-01-27

**Authors:** Jeong-Min Hwang, Ji-Hye Lee, Jung-Yong Yeh

**Affiliations:** 1 Veterinary Research Center, Green Cross Veterinary Products Co., Ltd., 227-5, Kugal-dong, Giheung-gu, Yongin-si, Gyeonggi-do 446-903, Republic of Korea; 2 Foreign Animal Diseases Division, Animal, Plant, and Fisheries Quarantine and Inspection Agency, Anyang-ro 175, Manan-gu, Anyang-si, Gyeonggi-do 430-824, Republic of Korea

## Abstract

**Background:**

During the routine laboratory cultivation of *Lawsonia intracellularis*, *Mycoplasma *contamination has been a frequent problem. When *Mycoplasma *contamination occurs in laboratories that study *L. intracellularis*, the cultures must be discarded for 4 reasons: 1) *Mycoplasma *is inevitably concentrated along with *L. intracellularis *during the passage of *L. intracellularis*; 2) *Mycoplasma *inhibits the growth of *L. intracellularis*; and 3) it is impossible to selectively eliminate *Mycoplasma *in *L. intracellularis *cultures. In this study, we observed the contamination of *Mycoplasma *species during *L. intracellularis *cultivation among multiple laboratories.

**Results:**

The presence of a *Mycoplasma *infection in the *L. intracellularis *cultures was verified using polymerase chain reaction (PCR), and a sequence analysis of the partial 16S rRNA and 23S rRNA genes was performed. A PCR-based assay using genus-specific universal primers revealed that 29 (85.3%) of the 34 cultures were contaminated with *Mycoplasma*, including 26 with *M. hyorhinis *(89.2%), 2 with *M. orale *(6.9%), and 1 with *M. fermentans *(3.4%). The *Mycoplasma *contamination was not the result of infection with material of pig origin. McCoy cells, which are required for the cultivation of *L. intracellularis*, were also ruled out as the source of the *Mycoplasma *contamination.

**Conclusions:**

In this study, *M. hyorhinis *was identified as the most common mollicute that contaminated *L. intracellularis *cultures. Whether *L. intracellularis *enhances the biological properties of *Mycoplasma *to promote infection in McCoy cells is not known. Because the McCoy cell line stocks that were used simultaneously were all negative for *Mycoplasma*, and the same worker handled both the McCoy cells to maintain the bacteria and the *L. intracellularis *cultures, it is possible that the *L. intracellularis *cultures are more vulnerable to *Mycoplasma *contamination. Taken together, these results suggest that continuous cultures of *L. intracellularis *must be tested for *Mycoplasma *contamination at regular intervals.

The GenBank accession numbers for the sequences reported in this paper are JN689375 to JN689377.

## Background

Mycoplasmas, which are bacteria that lack a cell wall and belong to the class *Mollicutes*, are found in the physiological flora of both humans and animals, can act as opportunistic pathogens, and are frequent contaminants of cell cultures. Because they frequently contaminate both primary and stable cell lines, species of the genera *Mycoplasma *and *Acholeplasma *are a major concern with regard to the regulatory aspects of biosafety [1-6]. *Mycoplasma *contamination affects many aspects of cell culture, which can lead to unreliable experimental results and potentially harmful biological products [7,8]. Undetected mycoplasmas can persist in a culture because unlike other bacteria and fungi, mollicute contaminants do not result in turbid growth or cause cell damage [9]. Moreover, the majority of mollicutes are resistant to the antibiotics that are commonly used in long-term cell cultures [10]. Of the 20 mollicute species that are known to cause cell culture contamination, 5 (*M. arginini*, *M. fermentans*, *M. hyorhinis*, *M. orale*, and *A laidlawii*) account for approximately 95% of contamination cases [11,12].

*Lawsonia intracellularis *is a highly fastidious, obligate intracellular, Gram-negative bacterium and is a relatively newly identified member of the Desulfovibrionaceae family [13]. *L. intracellularis *is one of the most prevalent enteric pathogens in pigs, where it causes acute intestinal hemorrhaging (proliferative hemorrhagic enteropathy, or PHE) in naïve adult pigs and a wasting disease (porcine intestinal adenomatosis, or PIA) in growing pigs [14]. The in vitro culture of *L. intracellularis *is only possible when the bacterium is co-cultivated with a range of cell lines [15,16]. Currently, most researchers prefer to use McCoy mouse fibroblast cells for the isolation and maintenance of *L. intracellularis *in vitro [17-23].

Cell culture has become an indispensable tool for studying proliferative enteropathy in horses and pigs; however, during the routine cultivation of *L. intracellularis *in the authors' laboratories, *Mycoplasma *contamination has been a serious and frequent problem. One widely used method to passage *L. intracellularis *cultures involves concentrating the bacteria that are extracted from infected cell monolayers [16,24]. One caveat to this method is that it has the potential to concentrate mycoplasmal agents in addition to *L. intracellularis*, and *Mycoplasma *contamination may have already occurred at the start of the *L. intracellularis *culture. It is inevitable that *Mycoplasma *is concentrated along with *L. intracellularis *during this culture process. We observed that *Mycoplasma *inhibited the growth of *L. intracellularis*, which was then eventually lost from the culture. If *Mycoplasma *contamination of *L. intracellularis *cultures occurs during cell culture maintenance, these cultures must be discarded because the selective elimination of *Mycoplasma *from *L. intracellularis *cultures is not possible.

In the Republic of Korea (RO Korea), 5 laboratories handling *L. intracellularis *have encountered *Mycoplasma *contamination during the process of maintaining this bacterium. *Mycoplasma *contamination is one of the most critical factors that determines the success of *L. intracellularis *cultivation and has remained one of the biggest obstacles in isolating *L. intracellularis*. However, previous studies have not examined this contamination issue; therefore, we describe here the profiles of bacterial contamination with various *Mycoplasma *species in cells that are used during *L. intracellularis *cultivation in multiple laboratories.

## Methods

### Isolates and laboratories

The isolates from each laboratory were originally derived from two low-passage-number clinical isolates. First, the PHE/KK421 strain of *L. intracellularis *was isolated from a finisher pig with acute PHE in 2002 [23], and the second strain (PIA/MyCoyL1), was isolated from a grower pig with PIA in 2010 [25]. Contamination was not observed in either the McCoy cells that were used to maintain *L. intracellularis *or in *L. intracellularis *cultures, which were distributed to each laboratory, at passage numbers 1 through 10. The isolates were maintained by different researchers in each of the 5 laboratories, which were all located in the RO Korea. The laboratories were not linked; each laboratory independently maintained its own isolate (PHE/KK421 and/or PIA/MyCoyL1). The specimens used in this study were collected from cultures that had been maintained by different laboratory workers in different laboratories.

### Test samples and DNA isolation

The test samples were chosen based on the fact that they were independently handled by different workers and were stored at different times. These samples were collected from *L. intracellularis *cultures that were not subjected to normal maintenance procedures because of suspected *Mycoplasma *contamination. Aliquots of frozen stocks from 2 or 3 passages of each culture were screened for the presence of mollicutes. The *Mycoplasma *contamination status of 34 *L. intracellularis *culture stocks was analyzed using polymerase chain reaction (PCR). *Mycoplasma *PCR testing was performed at the Animal, Plant, and Fisheries Quarantine and Inspection Agency in Anyang-si, RO Korea. Briefly, 500-μl aliquots of the *L. intracellularis *culture stocks were placed in microcentrifuge tubes for PCR analysis. These aliquots were concentrated via centrifugation at 13,000 × *g *for 10 min at room temperature followed by the removal of 480 μl of the medium and the addition of 380 μl of sterile water to the remaining 20 μl of the medium and the pellet. The resulting supernatants were discarded, and the pellets were washed twice with phosphate-buffered saline (PBS). After the second wash, the pellets were resuspended in 400 μl of PBS and were incubated for 15 min at 95°C. An automated DNA extraction was performed using a BioRobot M48 workstation apparatus (Qiagen, GmbH, Hilden, Germany) with a MagAttract Virus Mini M48 kit (Qiagen). Nucleic acids were recovered in 50 μl of the elution buffer. The eluted genomic DNA was stored at-70°C until use. The McCoy cell line stocks that were used simultaneously were also tested to determine whether the McCoy cells were the source of the *Mycoplasma *contamination.

### Mycoplasma PCR testing

To detect *Mycoplasma *DNA and to identify the contaminating *Mycoplasma *spp., 15 primers were used to amplify a conserved spacer region that encompasses the 16S and 23S rRNA genes as previously described (Table 1). *Mycoplasma *genus-specific PCR analysis was performed with a total reaction mixture volume of 20 μl. The 16S-23S rRNA regions of all mollicutes species that were used in the study were amplified using multiple primer sets under each PCR condition. The PCR conditions and oligonucleotide primers that were used to amplify the nucleic acids are shown in Table 1. The reactions were prepared in a volume of 25 μl that contained 2 μl of DNA, 1 U of Taq DNA polymerase, 250 μmol/l of each dNTP, 50 mmol/l of Tris-HCl (pH 8.3), 40 of mmol/l KCl, 1.5 mmol/l of MgCl_2_, gel loading dye, and 5 pmol of each primer set using a PCR master mix (AccuPower PCR PreMix, Bioneer Corp., Daejeon, RO Korea). To prevent amplicon contamination, aerosol-barrier tips were used. The reagent mixtures were prepared in a limited-access PCR clean room, were moved to a sample preparation room for the addition of cell lysates, and were finally moved to a PCR instrumentation room for amplification. The PCR amplifications were performed using an Eppendorf Mastercycler gradient thermal cycler (Eppendorf, Hamburg, Germany). A negative control with no template was also included. Thirteen strains of *Mycoplasma *were obtained from the American Type Culture Collection (ATCC, Edinburg, VA, US) for use as positive controls. These strains included *M. arthritidis *ATCC 13988, *M. bovis *ATCC 25025, *M. fermentans *ATCC 19989, *M. genitalium *ATCC 33530, *M. hominis *ATCC 23114, *M. hyorhinis *ATCC 17981, *M. neurolyticum *ATCC 15049, *M. orale *ATCC 23714, *M. pirum *ATCC 25960, *M. pneumoniae *ATCC 15531, *M. pulmonis *ATCC 23115, *M. salivarium *ATCC 23064, and *Ureaplasma urealyticum *ATCC 27618. The amplified products were analyzed on a 1.5% agarose gel containing 0.5 μg/ml ethidium bromide and were visualized at the maximum levels of intensity using a Gel Doc 1000 gel analysis system (Bio-Rad Laboratories, Hercules, CA, USA).

**Table 1:** PCR primer sequences

Target gene	Primer	Nucleotide sequence (5'→3')^a^	Reference
16S rRNA	MyU 5-1	CGCCTGAGTAGTACGTTCGC	[26]
	MyU 5-2	CGCCTGAGTAGTACGTACGC	
	MyU 5-3	TGCCTGAGTAGTACATTCGC	
	MyU 5-4	TGCCTGGGTAGTACATTCGC	
	MyU 5-5	CGCCTGGGTAGTACATTCGC	
	MyU 5-6	CGCCTGAGTAGTATGCTCGC	
	MyU 3-1^b^	GCGGTGTGTACAAGACCCGA	
	MyU 3-2^b^	GCGGTGTGTACAAAACCCGA	
	MyU 3-3^b^	GCGGTGTGTACAAACCCCGA	
16S and 23S rRNA	F1 (OF)	ACACCATGGGAGYTGGTAAT	[27]
	R1^b ^(OF)	CTTCWTCGACTTYCAGACCCAAGGCAT	
	FN2 (IF)	ACCTCCTTTCTACGGAGTACAA	
	R2^b ^(IR	GCATCCACCAWAWACYCTT	
	FN3 (IF)	TATTTGCTATTCAGTTTTCAAAGAAC	
	RN3^b ^(IR)	GGGGTGAAGTCGTAACAAGGTAT	

^a ^Abbreviations represent a mixed-base code, which was used to reduce the number of primers. Y = C, T and W = A, T

^b ^Primers are in the reverse orientation

### Sequencing of PCR products

The PCR products of the *Mycoplasma *spp. from the contaminated *L. intracellularis *cultures were purified using the ExoSAP-IT reagent (USB Corporation, Cleveland, OH, USA) to remove any unwanted deoxynucleoside triphosphates and primers from the PCR product mixture. A 2- μl aliquot of the clean PCR products was analyzed for purity and concentration on a 1% agarose gel, and then the remaining sample was cloned into the pGEM-T Easy Vector System I (Promega, Madison, WI, USA). We verified that the clones contained inserts by digesting the plasmid DNA with EcoRI (New England Biolabs, Hitchin, Hertfordshire, UK) and by separating the plasmid fragments on a 1.5% agarose gel. Sequencing analysis was performed by Macrogen, Inc. (Seoul, RO Korea). All of the sequences were identified using the BLAST search engine (http://blast.ncbi.nlm.nih.gov/Blast.cgi).

### Mycoplasma enrichment culture

To determine if M*ycoplasma *might be present with a titer below the detection limit of the PCR assays and to rule out the possibility of a false negative result, the samples were taken into culture and tested again over several passages. To enrich any *Mycoplasma *that might be present, the culture supernatant was incubated at 37°C in a controlled environment using pleuropneumonia-like organisms (PPLO) media, PPLO broth (21 g/l; Difco, Sparks, MD, USA), or PPLO agar (35 g/l; Difco), which consisted of 100 ml/l (25%) Baker's extract solution, 200 ml/l horse serum (Cambrex, USA), 2 ml/l (1%) phenol red (Sigma, USA), 5 ml/l (2.5%) thallium acetate (Sigma), 10 ml/l (106 U/l) penicillin G (Sigma), and a carbon source (either 10 g/l glucose, 2 g/l arginine, or 0.4 g/l urea). Either an anaerobic jar or a CO_2 _incubator was used for these experiments. Because bacterial metabolism leads to a pH shift, a sequential color change of the PPLO medium from results, going from red to orange and then to yellow. When the color change indicated that the bacteria were metabolically active, the culture solutions were plated onto PPLO agar plates. Colonies were evaluated by inverted microscopy every week for 2-3 weeks to determine the number of colony-forming units (CFU) and to monitor the development of typical colonies (a fried-egg appearance). *Mycoplasma *culture solutions were stored at -70°C.

### Investigation of indicator cell lines

Culture supernatants were transferred to a *Mycoplasma *negative indicator cell line such as Mardin-Darby Bovine Kidney (MDBK) and Vero and tested after a culturing period. Parallel cultures without supernatant were cultured concurrently as a negative control.

## Results

The contaminated cultures in this study had been produced between 2002 and 2010 in 5 different research laboratories that handled *L. intracellularis*. Aliquots from 29 of the 34 (85.3%) *L. intracellularis *cultures that contained an unidentified contaminant tested positive for *Mycoplasma *DNA (Table 2). The contaminant in the remaining 5 cultures remained unidentified because it was not amplified by the *Mycoplasma *PCR assay that was employed in this study. The results of each PCR assay were identical. To identify the *Mycoplasma *spp. in the contaminated cell culture samples, *Mycoplasma *DNA amplicons from selected positive cultures were sequenced and compared with the GenBank database and the database of the National Center for Biotechnology Information (NCBI, Bethesda, MD, USA). The majority of the *L. intracellularis *cultures with unidentified contamination were chronically infected with *Mycoplasma*. A total of 26 *L. intracellularis *cultures contained DNA sequences that matched those of *M. hyorhinis *(100% sequence identity), whereas 2 cultures and 1 culture contained DNA sequences that matched those of *M. orale *and *M. fermentans*, respectively (100% sequence identity). Each culture was infected with only a single species of mollicute. The stock specimens were deposited at intervals of 2 or 3 passages for each culture. The McCoy cell line stocks that were used simultaneously were all negative for *Mycoplasma*. These results suggest that McCoy cells, which are required for the cultivation of *L. intracellularis*, were not the source of the *Mycoplasma *contamination. The *M. orale*, *M. fermentans, and M. hyorhinis *16S and 23S rRNA sequences that were determined in this study have been deposited in the GenBank database under accession numbers JN689375 to JN689377. When the supernatant from the *Mycoplasma*-contaminated *Lawsonia *cultures was transferred to a *Mycoplasma*-negative indicator cell line (MDBK and Vero) and tested after an appropriate culture period, the indicator cell lines were infected with the same *Mycoplasma *species that were involved in the *L. intracellularis *infections. All parallel cultures that did not contain supernatant were *Mycoplasma *negative, and all of the PCR-negative samples were also confirmed to be negative when they were cultured and retested over the course of several passages.

**Table 2:** Mollicute strains identified in the *L.intracellularis *stock specimens

Laboratory^a^	Period stocked (years)	Isolate used^b ^(no. of passages, range)	# of Tested Cultures	# of *Mycoplasma*-Contaminated	*Mycoplasma *Spp. Identification (#)		
					
					*M. fermentans*	*M. hyorhinis*	*M. orale*
KKU	2002-2004	KK421 (20-30)	3	3		3	
NIAST	2004-2005	KK421 (20-30)	4	4		4	
NVRQS TSE	2005-2006	KK421 (40-70)	13	11	1	9	1
QIA EID	2006-2011	KK421 (20-30) and MyCoyL1 (10-20)	9	8		7	1
GC	2004-2011	KK421 (40-50) and MyCoyL1 (10-20)	5	3		3	

Total			34^c^	29	1	26	2

^a ^Abbreviation of the institutes in the Republic of Korea (RO Korea): KKU: Konkuk University, Seoul, RO Korea; NIAST: National Institute of Agricultural Sciences and Technology, Suwon-si, RO Korea; NVRQS: National Veterinary Research and Quarantine Service, Anyang-si, RO Korea; QIA: Animal, Plant and Fisheries Quarantine and Inspection Agency, Anyang-si, RO Korea; and GC: Green Cross Veterinary Products Co., Ltd., Yongin-si, RO Korea

^b ^A strain of *L. intracellularis *called the PHE/KK421 strain was isolated from a finisher pig with acute proliferative hemorrhagic enteropathy in 2002 [23]. A second strain called PIA/MyCoyL1 was isolated from a grower pig with porcine intestinal adenomatosis in 2010 [25]

^c ^The 5 samples were not contaminated with *Mycoplasma *and it was presumed that those contaminations were due to other unidentified microorganisms

## Discussion

Mollicute strains, including *Mycoplasma*, are widespread human, animal, and plant parasites [3]. In this study, the majority of the *Mycoplasma *contaminants in *L. intracellularis *cultures were of the species *M. hyorhinis*, which is one of the most commonly identified contaminating *Mycoplasma *species in laboratory cell culture [28,29], although it is a porcine respiratory parasite and is rarely found in human subjects. Routine screenings have now been conducted on a regular basis since this study was initiated in laboratories employed in this study. Chronic contamination with *M. hyorhinis *was detected in several cases, which resulted in the loss of the contaminated *L. intracellularis *cultures. Transient *Mycoplasma *contamination of *L. intracellularis *cultures was also detected in several cases during the *Mycoplasma *screening effort. Although these *L. intracellularis *cultures were maintained normally, and no cytopathic effects were seen in the McCoy cells, we confirmed that these cultures were indeed positive for *Mycoplasma *DNA. These cultures were defined as transiently contaminated because the infection no longer remained after two or three passages following the initial detection of *M. fermentans *or *M. orale *DNA. These transient contaminations were detected by both of the PCR assays that were used in this study and lasted for one to two weeks, after which the cultures remained negative for *Mycoplasma *DNA over several passages. In addition, to rule out possibility of DNA contamination from the laboratory setting that could result in both false positives and false negatives, we performed another completely independent and sensitive *Mycoplasma *detection assay using MycoAlert (Cambrex, USA). The results of this assay confirmed those of the PCR testing. The transient contaminations were found to occur in two of the three laboratories that were involved in this study, and the transiently contaminated *Mycoplasma *spp. in those two labs were identified to be *M. orale *and *M. fermentans *instead of *M. hyorhinis*. We observed that the *M. hyorhinis *(26), *M. orale *(2), and *M. fermentans *(1) contaminations that are listed in Table 2 were persistent in all detected cases. When we assessed the persistence and transience of these Mycoplasma spp., *M. hyorhinis *contaminations were persistent in all detected cases, while *M. orale *and *M. fermentans *were transient in half of the initially detected cases. These finding suggest that *L. intracellularis *cultures might be more vulnerable to long-term *M. hyorhinis *contamination, while contamination with *M. orale *and *M. fermentans *might be shorter lasting. To our knowledge, no transient *Mycoplasma *contaminations of cell cultures have been described thus far, although Lemoine et al. (1987) previously reported that *Mycoplasma *contamination during pregnancy appears to be very transient and has no effect on the immediate neonatal pathology [30].

These results support the idea that *Mycoplasma *contamination is common in *L. intracellularis *cultures and/or in the cell lines that are used to grow this bacterium. However, whether *L. intracellularis *enhances the biological properties of *Mycoplasma *to promote its infection of McCoy cells remains unclear. Because the McCoy cell line stocks that were used simultaneously were all negative for *Mycoplasma *contamination and the same worker handled both the McCoy cells and the *L. intracellularis *cultures, it appears that the *L. intracellularis *cultures may be more vulnerable to *Mycoplasma *contamination. In other words, if the person who handled both sets of cells was infected with *Mycoplasma*, this suggests that the *L. intracellularis *cultures were more vulnerable to *Mycoplasma *contamination for the following reasons: a) the *Mycoplasma *contamination did not stem from the original McCoy cell line; b) the *L. intracellularis *cultures were *Mycoplasma*-free for their first 10 passages prior to their distribution to each laboratory; c) handling the McCoy cell lines and the *L. intracellularis *was performed separately but by the same person. In other words, the *Mycoplasma *contamination was most likely introduced by the persons who handled both the McCoy cells and the *L. intracellularis *bacterial cultures; d) although it is a common problem in instances of mycoplasmal contamination, any cross-contamination of other cell lines, equipment, media, reagents, etc. in the laboratories could be ruled out because separate reagent containers were used in all cases (including bacterial cultures, McCoy cell cultures, and other cell lines); and e) to reduce cross-contamination in the laboratory setting, equipment was not shared by the bacterial and cell cultures. However, further studies are needed to determine the specific factors that are involved in the establishment of transient and chronic *Mycoplasma *contamination in *L. intracellularis *cultures.

Mycoplasmas are likely to be acquired by a host through intimate contact that results in the transfer of material between mucosal surfaces [31]; therefore, we cannot rule out the possibility that laboratory workers may be sources of *Mycoplasma *contamination in the cell cultures that they maintain. We do not believe that the contamination observed in this study occurred because of the use of contaminated cell lines or the presence of *Mycoplasma *spp. in the original specimens because we confirmed that the *L. intracellularis *culture were *Mycoplasma*-free for their first 10 passages before being distributed to each laboratory. Cross-contamination between the laboratories that participated in the present study can also be excluded because the laboratories were not linked and each laboratory independently grew and maintained its own isolate (PHE/KK421 or/and PIA/MyCoyL1), as described in the Materials and Methods. Taken together, these observations imply that the initial *Mycoplasma *contamination originated from human handling. These results suggest that continuous cultures of *L. intracellularis *must be tested for *Mycoplasma *contamination at regular intervals.

In the case of other intracellular bacteria, Krausse-Opatz et al. found that *M. fermentans*, *M. hyorhinis*, and *M. hominis *were present as contaminating agents [32]. Huniche et al. reported that 3 of 6 *Chlamydia pneumoniae *isolates from ATCC along with two Finnish isolates of that bacterium were infected with *M. hominis *and *M. orale *[33]. Although *M. hyorhinis *was found the most frequently in *L. intracellularis *cultures in this study, *M. hominis *has been more frequently reported in *Chlamydia *cultures in previous publications. Messmer et al. has also suggested that intracellular bacteria such as *Chlamydia *should be periodically screened for *Mycoplasma *contamination [34]. Careful attention is important in research that involves intracellular bacteria because *Mycoplasma *contamination could pose a serious problem for this kind of experiments.

## Conclusions

In this study, *M. hyorhinis *was identified as the most common mollicute that contaminated *L. intracellularis *cultures; however, it is still unclear whether *L. intracellularis *enhances the biological properties of *Mycoplasma *to promote the infection of the McCoy cells. Because the McCoy cell line stocks that were used simultaneously were all negative for *Mycoplasma*, and the same worker handled both the McCoy cells and the *L. intracellularis *cultures, this suggests that *L. intracellularis *cultures may be more vulnerable to *Mycoplasma *contamination. Taken together, these results suggest that continuous cultures of *L. intracellularis *must be tested for *Mycoplasma *contamination at regular intervals.

## Abbreviations

PHE: Proliferative hemorrhagic enteropathy; PIA: Porcine intestinal adenomatosis; RO Korea: Republic of Korea; PCR: Polymerase chain reaction; PBS: Phosphate-buffered saline.

## Competing interests

The authors declare that they have no competing interests.

## Authors' contributions

JYY conceived of the study, designed the experiments, wrote the manuscript, and finalized the manuscript. JMH and JHL conducted the majority of the experiments including the cultivation of *L. intracellularis *and McCoy cells, mycoplasma PCR, and sequencing analysis. All authors read and approved the final manuscript.
